# Risk and all-cause mortality of high low-density lipoprotein cholesterol-albumin ratio level in stable coronary artery disease patients following percutaneous coronary intervention

**DOI:** 10.3389/fendo.2026.1755378

**Published:** 2026-02-16

**Authors:** Qianyin Huang, Yunfeng Xia, Chenzhu Zhang, Minghua Lv, Qiongyin Liang, Zixuan Zeng, Kaixin Huang, Yuxuan Long, Xiaoyan Shi, Jingwen Zhuo, Erlin Zhou, Kaijun Xing, Zhuolun Li, Huifan Qiu, Jintong Pan, Hoi-kan Lee, Shenheng Li

**Affiliations:** 1 Department of Nephrology, Southern Medical University, Zhujiang Hospital, Guangzhou, Guangdong, China; 2 Department of Nephrology, The First Affiliated Hospital of Chongqing Medical University, Chongqing, China; 3 Medicine & Geriatrics, Tuen Mun Hospital, Hong Kong, Hong Kong SAR, China; 4 The Second Clinical Medical School of Southern Medical University, Guangzhou, Guangdong, China; 5 School of Public Health, Southern Medical University, Guangzhou, Guangdong, China

**Keywords:** albumin, all-cause mortality, coronary artery disease, LDL-C, percutaneous coronary intervention, ratio

## Abstract

**Background:**

Both low-density lipoprotein cholesterol (LDL-C) and albumin are well-established predictors of adverse outcomes in patients with stable coronary artery disease (CAD) who have undergone percutaneous coronary intervention (PCI). However, the risk and all-cause mortality (ACM) of the LDL-C-albumin ratio (LAR)—a composite index integrating these two parameters—remains unclear.

**Methods:**

This study enrolled 198 consecutive patients with newly diagnosed stable CAD who underwent PCI at Shinonoi General Hospital between 2014 and 2017. The primary aim was to assess the risks related to high LAR levels and the association between LAR and ACM in this population.

**Results:**

The association between the LAR and ACM was evaluated through multiple methods. Analyzed as a continuous variable, each 1 mg/g increase in LAR conferred a 6% higher adjusted risk of ACM (Hazard Ratio [HR] 1.06, 95% Confidence Interval [95% CI] 1.00–1.12, *P* = 0.035). When categorized into quartiles (Q1-Q4) based on LAR level, the highest LAR quartile (Q4) shows a statistically significant 3.72–4.01-fold increase in ACM risk (vs. Q1) after covariate adjustment, the risk is not linear across all groups but is concentrated in the top 25% of patients with the highest LAR values. This finding was reinforced by an inverse probability of treatment weighting (IPTW) model, which demonstrated that patients with a high LAR (≥ 32.5 mg/g) had a 3.16-fold greater risk of ACM than those with a low LAR (95% CI 1.25–7.97, *P* = 0.028). Four-year Kaplan-Meier analysis confirmed that the high LAR group had a significantly lower adjusted survival probability (*P* = 0.017), with adjustments made for the same variables as those included in the IPTW analysis. In a separate analysis, LAR ≥ 32.5 mg/g is driven by elevated levels of atherogenic lipids (LDL-C, TC) and reduced serum albumin. Notably, the protective effect of male sex on high LAR-related risk requires further investigation.

**Conclusions:**

High level LAR is associated with ACM in stable CAD patients following PCI, with a level above 32.5 mg/g distinguishing a high-risk subgroup that warrants closer clinical monitoring due to a significantly elevated risk of adverse outcomes.

## Introduction

It is widely accepted that elevated LDL-C levels constitute a major risk factor for coronary heart disease. However, even patients who have achieved LDL-C levels below the currently recommended targets may still be at residual cardiovascular risk (1, 2). Among patients who PCI, those who achieved a 50% or greater reduction in LDL-C levels experienced a decreased risk of major adverse cardiovascular and cerebrovascular events, irrespective of their baseline LDL-C levels (3). While achieving an LDL-C reduction of 50% or more remains a critical therapeutic goal, targeting LDL-C levels below 1.4 mmol/L after PCI may confer additional clinical benefits (3). In contrast, among patients who underwent PCI for in-stent restenosis lesions, LDL-C levels were not associated with adverse prognostic outcomes (4). Although the addition of ezetimibe to atorvastatin can reduce LDL-C and high-sensitivity C-reactive protein (CRP) levels in short-term follow-up, it does not effectively lower short-term major cardiovascular events in post-PCI patients; thus, studies with longer-term follow-up are recommended (5). Data from the ChinaHEART cohort shows Each 1 mmol/L increase in total cholesterol (TC), LDL-C, and non-high-density lipoprotein cholesterol (non-HDL-C) was associated with multivariable-adjusted HRs of 1.16 (95% CI 1.10,1.22), 1.23 (1.15,1.32) and 1.16 (1.10,1.23) for ACM, respectively (6). Notably, these individuals often achieve optimal LDL-C levels while displaying elevated apolipoprotein B and non-HDL-C concentrations, which highlights the limitations of using LDL-C as the sole marker for atherosclerotic cardiovascular disease risk stratification (7).

On the other hand, low serum albumin levels independently predicted major adverse cardiovascular events in patients undergoing PCI (8, 9). Analysis from a real-world retrospective cohort study indicated that the CRP-albumin ratio is an independent and novel predictor of long-term adverse outcomes in CAD patients who have undergone PCI (10), with a higher CRP-albumin ratio being associated with poorer 5-year outcomes among diabetic patients who underwent PCI (11). A growing body of evidence has demonstrated that numerous albumin-related composite indicators are associated with ACM following PCI. These indicators include the fibrinogen-to-albumin ratio (12), red blood cell distribution width-to-albumin ratio (13), serum globulin-to-albumin ratio (14), and alkaline phosphatase-to-albumin ratio (15). In contrast, the relationship between the LAR and ACM in patients after PCI remains unclear to date.

To address this research gap, the present study aimed to introduce LAR as a novel composite indicator and investigate its association with ACM in stable CAD patients in the 4-year follow-up period after PCI. Furthermore, we employed IPTW analyses and multivariate Cox proportional hazards regression analyses to clarify the independent relationship between LAR and ACM in this specific patient population.

## Materials and methods

### Population characteristics

**Figure 1:**
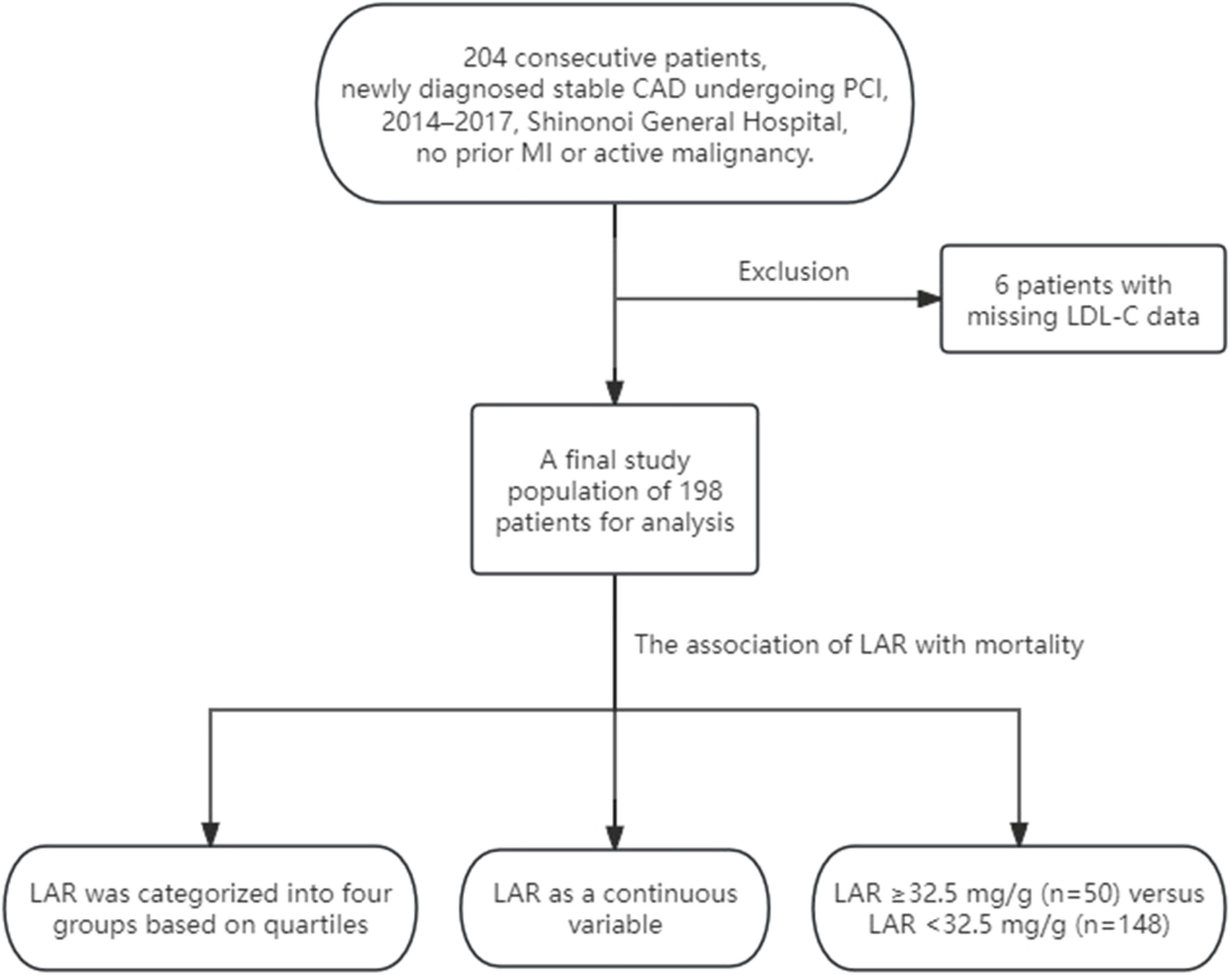
Flow chart for the study.

This retrospective, single-center cohort study enrolled 204 consecutive patients admitted to Shinonoi General Hospital between October 2014 and October 2017. This study included patients with newly diagnosed stable CAD (prior myocardial infarction excluded) who underwent elective PCI, while those with active malignancy were excluded (8). Six patients with missing LDL-C data were excluded from the full dataset, resulting in a final study population of 198 patients for analysis. **Figure 1** depicts the flow chart of the study. Enrollment followed approval by the Shinonoi General Hospital Ethics Committee and written informed consent from each participant.

The data used in this study were obtained from the open-access Dryad database (https://datadryad.org), where the original dataset is publicly available for download (https://doi.org/10.5061/dryad.fn6730j), and additional ethical approval and informed consent were not required for the present analysis. No data on dietary intake or eating habits were collected from the study participants. Blood samples in this study were collected after the initiation of lipid-lowering therapy. All data were fully anonymized prior to access and utilization.

### Definition of the high-level LAR and data collection

Because a high-risk subgroup was defined using the highest LAR quartile (Quartile 4 [Q4]: 37.0, Interquartile Range [IQR]: 33.4–40.5) as the threshold by multi-model Cox regression in this study, the LAR cutoff value of 32.5 mg/g was established based on clinically relevant thresholds for its individual components. This was derived by taking the recognized risk threshold for LDL-C (130 mg/dL) in patients with CAD (16) or the general population (17) and the established prognostic threshold for albumin (4.0 g/dL) (8) in CAD patients. The specific value was calculated as the ratio of these two thresholds: 130 mg/dL divided by 4.0 g/dL, resulting in 32.5 mg/g.

Stable CAD was defined as angiographic stenosis ≥ 90% in the epicardial coronary artery or angiographic stenosis ≥ 75% in the epicardial coronary artery with either a symptom of chest pain induced by exercise or evidence of stress-induced ischemia via any clinical stress testing modality (8, 18). Coronary angiography and PCI were performed, according to the guidelines and standard protocols. Investigators collected comprehensive data on enrolled patients, including clinical characteristics, medical history, major CAD-related risk factors, comorbidities, prescribed medications, and findings from laboratory tests, echocardiography, coronary angiography, discharge evaluations, and post-discharge follow-up. In addition to statins, three patients were also treated with ezetimibe, all of whom were distributed in the LAR < 32.5 mg/g group.

### Statistical analysis

Baseline characteristics stratified by the LAR cutoff value (32.5 mg/g) were compared using the chi-square test for categorical variables and the Mann-Whitney U test for continuous variables. Multiple Cox proportional hazards regression analysis was performed to evaluate LAR both as a continuous variable and a categorical variable (stratified by quartiles [Q1 to Q4]), with the objective of investigating its association with the adjusted risk of ACM in the total study population over a 4-year follow-up period. To identify informative and stable covariates for the multivariate model, we first evaluated the variance inflation factor (VIF) to assess multicollinearity (a VIF < 5 was defined as no significant multicollinearity). We then screened covariates based on three criteria: (1) no severe multicollinearity (VIF < 5); (2) meaningful contribution to the model (assessed via coefficient stability); (3) clinical relevance. Based on the aforementioned criteria, covariates including age, sex, body mass index (BMI), dyslipidemia (DLP), diabetes mellitus (DM), peripheral arterial disease (PAD), CRP, statin use, estimated glomerular filtration rate (eGFR), hypertension (HT), and old cerebral infarction (OCI) were all incorporated into the various models for analysis, and all these variables were additionally adjusted for in the multivariable logistic regression analyses. All candidate covariates exhibited a VIF < 5 (range: 1.319–2.311), indicating no severe multicollinearity in these models. Aspirin use was not included in the model analysis due to its widespread use among the study population. To evaluate the model’s performance, the goodness-of-fit was assessed using the Log-rank Test, which compared ACM risk between high- and low-risk groups. A *P*-value < 0.05 was considered to indicate effective stratification, confirming the model’s discriminative ability. IPTW analysis combined with adjusted Kaplan-Meier (K-M) curves was utilized to assess the ACM risk associated with LAR at the 32.5 mg/g cutoff. Missing values for CRP were present in 4% of cases (n = 8) and were imputed using simple mean imputation as part of the IPTW analysis. All variables included in the IPTW analysis exhibited an SMD < 0.1, indicating that covariate balance was adequately achieved. In the present study, to minimize bias, IPTW analysis, and adjusted K-M curves were all adjusted for the same set of covariates, namely age, sex, BMI, DLP, DM, PAD, CRP, and statin use. All statistical analyses were performed using R software version 4.2.1 (http://www.R-project.org; The R Foundation for Statistical Computing, Vienna, Austria) and Free Statistics software version 2.2 (Beijing FreeClinical Medical Technology Co., Ltd., Beijing, China).

## Results

### Baseline characteristics

**Tables 1**–**4** report the baseline characteristics of the study cohort (n = 198), which was stratified into two groups according to LAR levels: a low LAR group (LAR < 32.5 mg/g, n = 148) and a high LAR group (LAR ≥ 32.5 mg/g, n = 50). The median follow-up duration was 792 days, with a maximum follow-up period of 4 years. The lower LAR group had a significantly higher proportion of males (74.3% vs. 56.0%, *P* = 0.015) and a higher prevalence of hypertension (HT) (78.4% vs. 64.0%, *P* = 0.043). As expected from the LAR grouping, there were profound differences in the lipid profiles: the higher LAR group had significantly elevated levels of TC (median 210.0 vs. 175.0 mg/dL, *P* < 0.001) and LDL-C (median 143.0 vs. 100.0 mg/dL, *P* < 0.001). No significant differences were found in triglycerides (TG), HDL-C, or HbA1c. The higher LAR group had a significantly lower serum albumin level (median 3.9 vs. 4.1 g/dL, *P* = 0.042). There were no significant differences in estimated glomerular filtration rate (eGFR), liver enzymes (AST/ALT), inflammatory markers (CRP), left ventricular ejection fraction (LVEF), or complexity of coronary artery disease (e.g., multivessel PCI, bifurcation lesions, calcification). The higher LAR group had a numerically higher rate of ACM (16.0% vs. 6.8%, *P* = 0.083). These findings suggest that LAR may be associated with lipid metabolism and certain baseline clinical features, while its correlation with post-PCI outcomes requires further validation.

**Table 1:** Baseline clinical characteristics.

Variables	voverall population	LAR < 32.5 mg/g	LAR ≥ 32.5 mg/g	*P*-value
	Total (n = 198)	(n =148)	(n = 50)	
Baseline clinical characteristics				
Age (years)	72.5 (66.0, 80.0)	74.0 (67.0, 80.0)	69.0 (64.2, 79.0)	0.179
Male, n (%)	138 (69.7)	110 (74.3)	28 (56)	0.015*
BMI ( kg/m²)	23.5 (21.1, 25.7)	23.8 (21.3, 25.7)	22.6 (20.5, 25.0)	0.151
HT, n (%)	148 (74.7)	116 (78.4)	32 (64)	0.043*
sBP (mmHg)	136.5 (123.0, 147.0)	137.0 (122.8, 148.0)	136.0 (125.2, 146.8)	0.94
dBP (mmHg)	76.0 (69.2, 85.0)	76.0 (69.0, 85.0)	76.5 (70.2, 88.5)	0.274
DM, n (%)	70 (35.4)	53 (35.8)	17 (34)	0.817
DLP, n (%)	98 (49.5)	79 (53.4)	19 (38)	0.06
PAD, n (%)	53 (26.8)	38 (25.7)	15 (30)	0.55
OCI, n (%)	34 (17.2)	26 (17.6)	8 (16)	0.799
AF, n (%)	25 (12.6)	20 (13.5)	5 (10)	0.518
Past smoker, n (%)	97 (49.0)	75 (50.7)	22 (44)	0.414

Continuous variables: median (IQR); categorical variables: n (%). Mann-Whitney U test was used for the comparison of non-normally distributed continuous variables (presented as median (IQR)) between the two groups. Pearson’s chi-square test was used for the comparison of categorical variables between the two groups; Statistical significance was defined as: *P < 0.05, **P < 0.01, ***P < 0.001. LAR, LDL-C-albumin ratio; BMI, body mass index; HT, hypertension; sBP, systolic blood pressure; dBP, diastolic blood pressure; DM, diabetes mellitus; DLP, dyslipidemia; PAD, peripheral arterial disease; OCI, old cerebral infarction; AF, atrial fibrillation.

**Table 2:** Medication use.

Variables	overall population	LAR < 32.5 mg/g	LAR ≥ 32.5 mg/g	*P*-value
	Total (n = 198)	(n =148)	(n = 50)	
Medication use				
Aspirin, n (%)	196 (99.0)	146 (98.6)	50 (100)	1
Warfarin, n (%)	5 ( 2.5)	4 (2.7)	1 (2)	1
DOAC, n (%)	20 (10.1)	16 (10.8)	4 (8)	0.569
Thienopiridines, n (%)	194 (98.0)	144 (97.3)	50 (100)	0.574
PPI, n (%)	129 (65.2)	98 (66.2)	31 (62)	0.589
Statins, n (%)	108 (54.5)	77 (52)	31 (62)	0.221
ACEI, n (%)	18 ( 9.1)	15 (10.1)	3 (6)	0.57
ARB, n (%)	86 (43.4)	63 (42.6)	23 (46)	0.672
β-blocker, n (%)	54 (27.3)	39 (26.4)	15 (30)	0.616
MRA, n (%)	11 ( 5.6)	5 (3.4)	6 (12)	0.032*

Data are presented as n (%) for all categorical variables. Statistical significance was defined as: *P < 0.05. Fisher’s exact test was applied for Warfarin and MRA comparisons due to small expected cell counts in some subgroups; Pearson’s Chi-square test was used for all other medication variables.

**Table 3:** Laboratory and cardiac interventional data.

Variables	overall population	LAR < 32.5 mg/g	LAR ≥ 32.5 mg/g	*P*-value
	Total (n = 198)	(n =148)	(n = 50)	
Laboratory data				
Hb (g/dL)	13.9 (12.4, 15.0)	13.9 (12.5, 15.1)	13.4 (12.2, 14.7)	0.388
Albumin (g/dL)	4.0 (3.6, 4.3)	4.1 (3.7, 4.3)	3.9 (3.4, 4.3)	0.042*
HbA1c (%)	6.0 (5.7, 6.7)	6.0 (5.7, 6.7)	6.1 (5.6, 6.8)	0.889
eGFR (mL/min/1.73m^2^)	64.0 (52.2, 74.0)	63.5 (52.8, 74.2)	65.0 (52.2, 72.8)	0.668
ALT (U/L)	18.0 (14.0, 26.0)	18.0 (14.0, 26.0)	19.0 (11.2, 26.5)	0.617
AST (U/L)	22.0 (18.0, 29.0)	22.0 (18.0, 28.0)	22.5 (18.0, 29.0)	0.886
TC (mg/dL)	184.0 (165.2, 208.0)	175.0 (156.0, 192.0)	210.0 (201.0, 224.0)	< 0.001***
TG (mg/dL)	117.0 (84.0, 160.0)	111.0 (80.8, 157.5)	117.0 (85.0, 184.0)	0.531
HDL-C (mg/dL)	49.0 (41.0, 57.0)	50.0 (41.0, 57.5)	47.0 (41.0, 57.0)	0.471
LDL-C (mg/dL)	109.0 (90.0, 129.0)	100.0 (87.0, 115.2)	143.0 (124.0, 151.8)	< 0.001***
CRP (mg/dL)	0.1 (0.0, 0.3)	0.1 (0.0, 0.3)	0.1 (0.1, 0.4)	0.089
Cardiac lesions				
LVEF (%)	66.0 (62.0, 68.0)	66.7 (62.6, 68.1)	65.0 (61.1, 68.0)	0.135
Multivessel PCI, n (%)	51 (25.8)	36 (24.3)	15 (30)	0.428
DES, n (%)	187 (94.4)	139 (93.9)	48 (96)	0.773
Bifurcation, n (%)	99 (50.0)	71 (48)	28 (56)	0.326
LMT, n (%)	12 ( 6.1)	10 (6.8)	2 (4)	0.734
Ostial, n (%)	30 (15.2)	24 (16.2)	6 (12)	0.472
Calc, n (%)	27 (13.6)	21 (14.2)	6 (12)	0.697
CTO, n (%)	12 ( 6.1)	8 (5.4)	4 (8)	0.503

Continuous variables (laboratory data, left ventricular ejection fraction [LVEF]) were presented as median (interquartile range, IQR) and compared between groups via the Mann-Whitney U test. Categorical variables (e.g., cardiac lesion characteristics) were expressed as n (%), with group comparisons using the Pearson’s chi-square test. For subgroups with expected cell counts <5 (left main trunk [LMT], chronic total occlusion [CTO]), Fisher’s exact test was used for valid statistical analysis. Statistical significance was set at *P < 0.05.

**Table 4:** Clinical outcomes.

Variables	overall population	LAR < 32.5 mg/g	LAR ≥ 32.5 mg/g	*P*-value
	Total (n = 198)	(n =148)	(n = 50)	
Clinical outcomes				
ACM, n (%)	18 ( 9.1)	10 (6.8)	8 (16)	0.083
Cardiac death, n (%)	6 ( 3.0)	3 (2)	3 (6)	0.17
ACM-MI-Stroke, n (%)	28 (14.1)	19 (12.8)	9 (18)	0.365
Cardiac death-MI-Stroke, n (%)	19 ( 9.6)	15 (10.1)	4 (8)	0.786
MI, n (%)	3 ( 1.5)	2 (1.4)	1 (2)	1
Stroke, n (%)	11 ( 5.6)	10 (6.8)	1 (2)	0.296

Statistical significance: *P < 0.05; **P < 0.01. All outcomes are categorical (n/%) and compared between groups using Pearson’s Chi-square test (expected cell count ≥5) or Fisher’s exact test (expected cell count <5), consistent with baseline categorical variable analysis. MI, myocardial infarction.

### Association of ACM with key clinical variables

**Table 5** identifies key clinical variables associated with ACM. In this elderly post-PCI CAD cohort, patients who died were significantly more likely to be older (≥70 years), have reduced renal function (eGFR <60 mL/min/1.73m²), and have dyslipidemia. In contrast, a BMI ≥25 kg/m² was significantly less common among those who died, suggesting a potential “obesity paradox.” Other cardiovascular risk factors, including diabetes, hypertension, and prior cerebrovascular disease, were not significantly associated with mortality in this univariate analysis. These findings underscore the importance of focusing on lipid management and renal function monitoring to mitigate mortality risk in this population.

**Table 5:** Association of ACM with key clinical variables.

Variables	overall population	Non-ACM	ACM	*P*-value
	Total (n = 198)	(n =180)	(n = 18)	
Male, n (%)	138 (69.7)	126 (70)	12 (66.7)	0.769
Age ≥70 years, n (%)	120 (60.6)	104 (57.8)	16 (88.9)	0.01*
BMI ≥25 kg/m², n (%)	66 (33.3)	65 (36.1)	1 (5.6)	0.009**
DM, n (%)	70 (35.4)	66 (36.7)	4 (22.2)	0.222
HT, n (%)	148 (74.7)	135 (75)	13 (72.2)	0.78
DLP, n (%)	98 (49.5)	82 (45.6)	16 (88.9)	< 0.001***
PAD, n (%)	53 (26.8)	45 (25)	8 (44.4)	0.094
eGFR <60 mL/min/1.73m^2^, n (%)	77 (38.9)	65 (36.1)	12 (66.7)	0.011*
AF, n (%)	25 (12.6)	21 (11.7)	4 (22.2)	0.254
OCI, n (%)	34 (17.2)	30 (16.7)	4 (22.2)	0.52

Data are presented as n (%) for all categorical variables. Pearson’s chi-square test (expected cell count ≥5) or Fisher’s exact test (expected cell count <5) was used for comparison of categorical variables between the Non-ACM and ACM groups. Statistical significance was defined as: *P < 0.05; **P < 0.01; ***P < 0.001. ACM, all-cause mortality; BMI, body mass index; DM, diabetes mellitus; HT, hypertension; DLP, dyslipidemia; PAD, peripheral arterial disease; eGFR, estimated glomerular filtration rate; AF, atrial fibrillation; OCI, old cerebral infarction.

### Univariate and multivariate analysis of factors associated with LAR ≥ 32.5 mg/g

**Table 6** presents the unadjusted associations between various baseline factors and the presence of LAR ≥ 32.5 mg/g. The univariate analysis clearly shows that the elevated LAR is primarily driven by its biochemical components: high LDL-C and low albumin are the strongest and most significant predictors. The association with male sex and HT being protective factors is an interesting clinical finding that warrants further investigation. LVEF and DLP showed marginal trends toward association.

**Table 6:** Univariate logistic regression analysis of factors associated with LAR ≥ 32.5 mg/g.

Risk factors	Univariate		
	OR	95% CI	*P*-value
Characteristics			
Age	0.98	0.95-1.01	0.211
Male	0.44	0.23-0.86	0.016*
BMI	0.95	0.87-1.04	0.245
HT	0.49	0.24-0.99	0.045*
DLP	1.87	0.97-3.6	0.062
DM	0.92	0.47-1.81	0.817
AF	0.71	0.25-2.01	0.519
PAD	1.24	0.61-2.52	0.551
OCI	0.89	0.38-2.13	0.799
eGFR	1	0.98-1.01	0.726
LVEF	0.97	0.94-1	0.051
Laboratory data			
TC	1.04	1.03-1.06	<0.001***
LDL-C	1.08	1.06-1.11	<0.001***
TG	0.99	0.96-1.03	0.619
HDL-C	0.99	0.97-1.02	0.626
HbA1C	1.1	0.81-1.48	0.552
Hb	0.93	0.79-1.09	0.342
Albumin	0.42	0.23-0.78	0.005**
CRP	1.09	0.79-1.49	0.614
Cardiac lesions			
LMT	0.57	0.12-2.72	0.485
Ostial	0.7	0.27-1.84	0.474
Bifurcation	1.38	0.72-2.63	0.327
Calc	0.82	0.31-2.18	0.697
CTO	1.52	0.44-5.29	0.509

Statistical Significance Level:*P < 0.05; **P < 0.01; ***P < 0.001.

**Table 7** presents the results of a multivariate analysis identifying factors independently associated with LAR ≥ 32.5 mg/g, following adjustment for potential confounding variables (age, sex, BMI, HT, DM, DLP, PAD, OCI, eGFR, CRP and statins use). The multivariate analysis indicates that an elevated LAR is driven predominantly by its constituent parts, with higher LDL-C and TC levels increasing its likelihood, and higher albumin levels decreasing it. Furthermore, male sex was identified as an independent protective factor, even after adjustment for key covariates.

**Table 7:** Multivariate logistic regression analysis of factors associated with LAR ≥ 32.5 mg/g.

Risk factors	Multivariate		
	OR	95% CI	*P*-value
LDL-C	1.18	1.11-1.25	<0.001***
TC	1.05	1.03-1.07	<0.001***
Albumin	0.17	0.06-0.44	<0.001***
Male	0.4	0.19-0.86	0.019*

Statistical Significance Level: *P < 0.05; ***P < 0.001. Adjusted for age + sex + BMI + HT + DM + DLP + PAD + OCI + eGFR + Statins + CRP.

### Multi-model Cox regression for ACM: albumin and LAR as continuous variables

**Table 8** presents findings from Cox proportional hazards regression analyses investigating the associations of two continuous variables, albumin and LAR, with ACM. LAR shows a modest but increasingly significant positive association with ACM as models adjust for key covariates. The final model (IV) indicates that a higher LAR is associated with a 6% increased risk of death per 1 mg/g (HR 1.06, 95% CI 1.00–1.12, *P* = 0.035), independent of a comprehensive set of clinical variables. Albumin shows a strong, consistent, and statistically significant protective association with ACM across all models. In the fully adjusted model (Model IV), the Hazard Ratio (HR) is 0.11. This means a 1g/dL increase in albumin is associated with an 89% reduction in the risk of death.

**Table 8:** Multi-model Cox regression for ACM: Albumin and LAR as continuous variables.

Variable	n.total	n.event_%	Model I		Model II		Model III		Model IV	
			HR (95%CI)	*P*-value	HR (95%CI)	*P*-value	HR (95%CI)	*P*-value	HR (95%CI)	*P*-value
Albumin	198	18 (9.1)	0.07 (0.03-0.17)	<0.001	0.1 (0.04-0.23)	<0.001	0.1 (0.04-0.26)	<0.001	0.11 (0.04-0.29)	<0.001
LAR	198	18 (9.1)	1.04 (0.99-1.1)	0.11	1.06 (1.01-1.12)	0.015	1.07 (1.01-1.12)	0.014	1.06 (1-1.12)	0.035

Model I: crude Model; Model II: age + sex + DM; Model III: age + sex + DM + eGFR; Model IV: age + sex + BMI + DM + HT + eGFR. Covariates were selected to address the specific research question of this table.

### Multi-model Cox regression for ACM with the LAR as a four-category variable

**Table 9** presents sequentially adjusted Cox proportional hazards regression analyses evaluating the association between LAR stratified into four quartile categories (Q1-Q4, Q1 as reference) and ACM. LAR quartiles are defined with median (IQR) values, and four incrementally adjusted models (crude to fully adjusted) are used to assess the HR and statistical significance of each LAR quartile for ACM. The highest LAR quartile (Q4) shows a statistically significant 3.72–4.01-fold increase in ACM risk (vs. Q1) after covariate adjustment, and this risk elevation persists and is slightly strengthened in the fully adjusted model (Model IV). The findings demonstrate that a very high LAR is a powerful and independent risk factor for mortality. The risk is not linear across all groups but is concentrated in the top 25% of patients with the highest LAR values.

**Table 9:** Multi-model Cox regression for ACM with the LAR as a four-category variable (Q1-Q4).

Outcome	Groups	n.total	n.event_%	Model I		Model II		Model III		Model IV	
				HR (95%CI)	*P*-value	HR (95%CI)	*P*-value	HR (95%CI)	*P*-value	HR (95%CI)	*P*-value
ACM	Q1	50	5 (10)	1(Ref)		1(Ref)		1(Ref)		1(Ref)	
	Q2	48	2 (4.2)	0.45 (0.09-2.33)	0.341	0.58 (0.11-3.08)	0.525	0.59 (0.11-3.14)	0.536	0.54 (0.1-2.89)	0.475
	Q3	50	3 (6)	0.62 (0.15-2.6)	0.515	1.04 (0.23-4.76)	0.957	1.03 (0.22-4.75)	0.972	1.23 (0.26-5.82)	0.79
	Q4	50	8 (16)	1.68 (0.55-5.13)	0.366	3.75 (1.06-13.2)	0.04	3.72 (1.05-13.15)	0.041	4.01 (1.14-14.13)	0.03
	Trend test	198	18 (9.1)	1.25 (0.83-1.91)	0.288	1.63 (1.02-2.6)	0.039	1.63 (1.02-2.6)	0.042	1.69 (1.06-2.69)	0.028

Model I: crude Model; Model II: age + sex + BMI + DLP; Model III: age + sex + BMI + DM + DLP; Model IV: age + sex + BMI + DLP + DM + Statins. Covariates were selected to address the specific research question of this table. LAR quartile cutoffs (mg/g): Q1:20.7(18.1,22.2); Q2:25.4(24.7,26.3); Q3:29.4(28.5,30.5); Q4:37.0(33.4,40.5).

### IPTW analyses for ACM with the LAR cutoff of 32.5 mg/g

**Figure 2:**
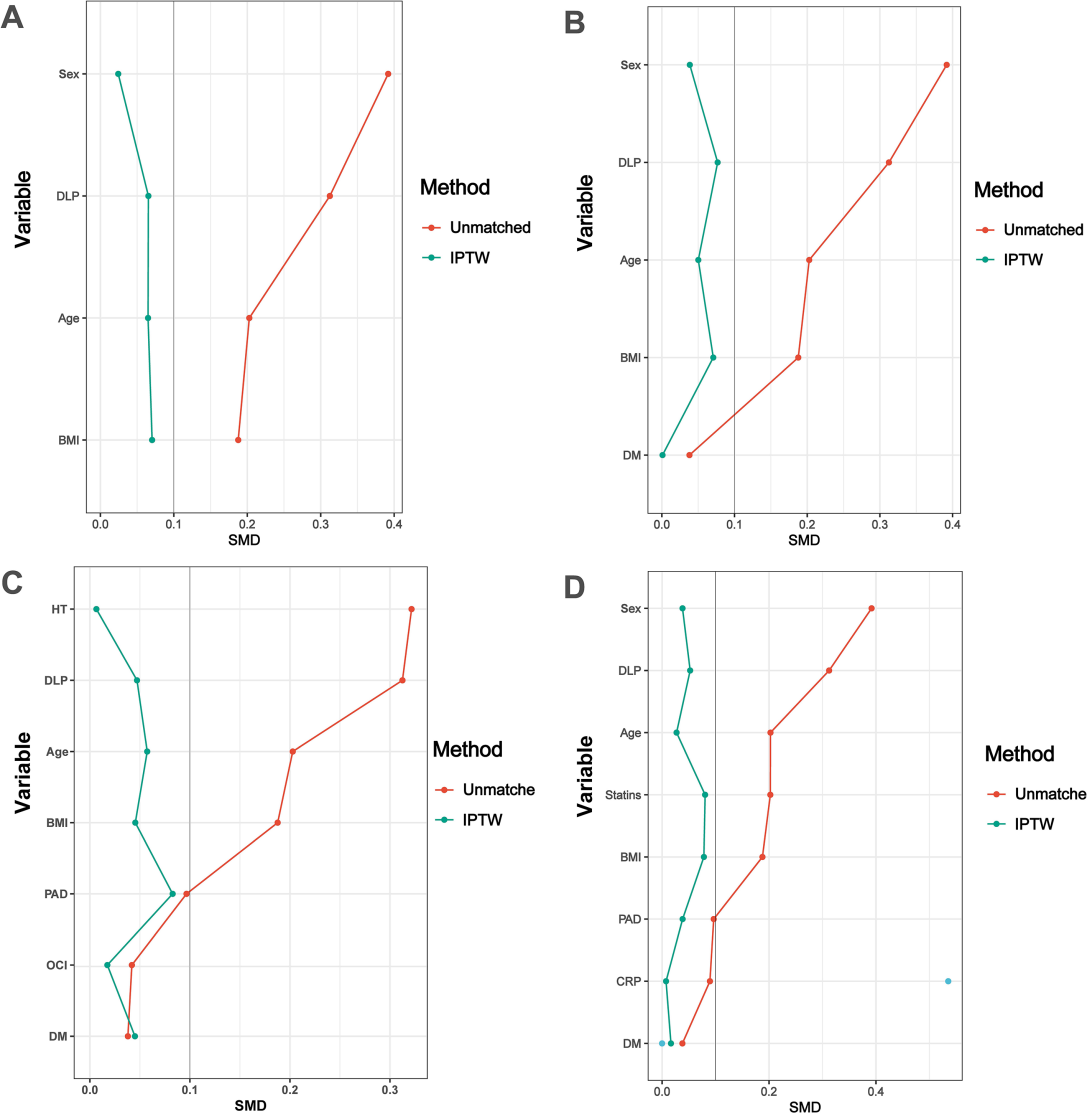
SMD values of variables in the IPTW analyses (**Table 10**) are less than 0.1. **(A)** Model I; **(B)** Model II; **(C)** Model III; **(D)** Model IV.

**Table 10** presents the results of sensitivity analyses using IPTW to estimate the association between a high LAR (≥ 32.5 mg/g) and the risk of ACM. The IPTW method creates a weighted pseudo-population where the measured baseline characteristics between the high and low LAR groups are balanced, simulating a randomized trial-like scenario. After balancing the groups for the fullest set of covariates (age, sex, BMI, DM, DLP, PAD, CRP, and Statins), patients with a high LAR (≥ 32.5 mg/g) had a 3.16-fold higher risk of ACM compared to those with a low LAR (95% CI 1.25–7.97, *P* = 0.028). The IPTW models were constructed by weighting patients based on their propensity scores to balance covariates with SMD < 0.1 (**Figures 2A–D**).

**Table 10:** IPTW analyses for ACM with the LAR cutoff of 32.5 mg/g in all cohort patients.

Outcomes	Sensitivity Analyses	Model I		Model II		Model III		Model IV	
		HR (95%CI)	*P*-value	HR (95%CI)	*P*-value	HR (95%CI)	*P*-value	HR (95%CI)	*P*-value
ACM	Unmatched	2.39 (0.94-6.06)	0.067	2.39 (0.94-6.06)	0.067	2.39 (0.94-6.06)	0.067	2.39 (0.94-6.06)	0.067
	IPTW	3.21 (1.26-8.17)	0.021	3.06 (1.19-7.83)	0.023	3.08 (1.24-7.61)	0.022	3.16 (1.25-7.97)	0.028

Variables in the IPTW analyses as follows. Model I: age + sex + BMI + DLP; Model II: age + sex + BMI + DM + DLP; Model III: age + BMI + DM + DLP + HT + OCI + PAD; Model IV: age + sex + BMI + DLP + DM + PAD + CRP + Statins. Variables with SMD value <0.1 in these sensitivity analyses. Covariates were selected to address the specific research question of this table. Across the three matched analyses (IPTW), the number of endpoint events is presented as follow: Model II 16.33 % (8/49) vs 11.22 % (11/98); Model III 16.33 % (8/49) vs 9.8 % (10/98) and Model IV 16 % (8/50) vs 10 % (10/100).

### Adjusted Kaplan-Meier curves for ACM with the LAR cutoff of 32.5 mg/g

**Figure 3:**
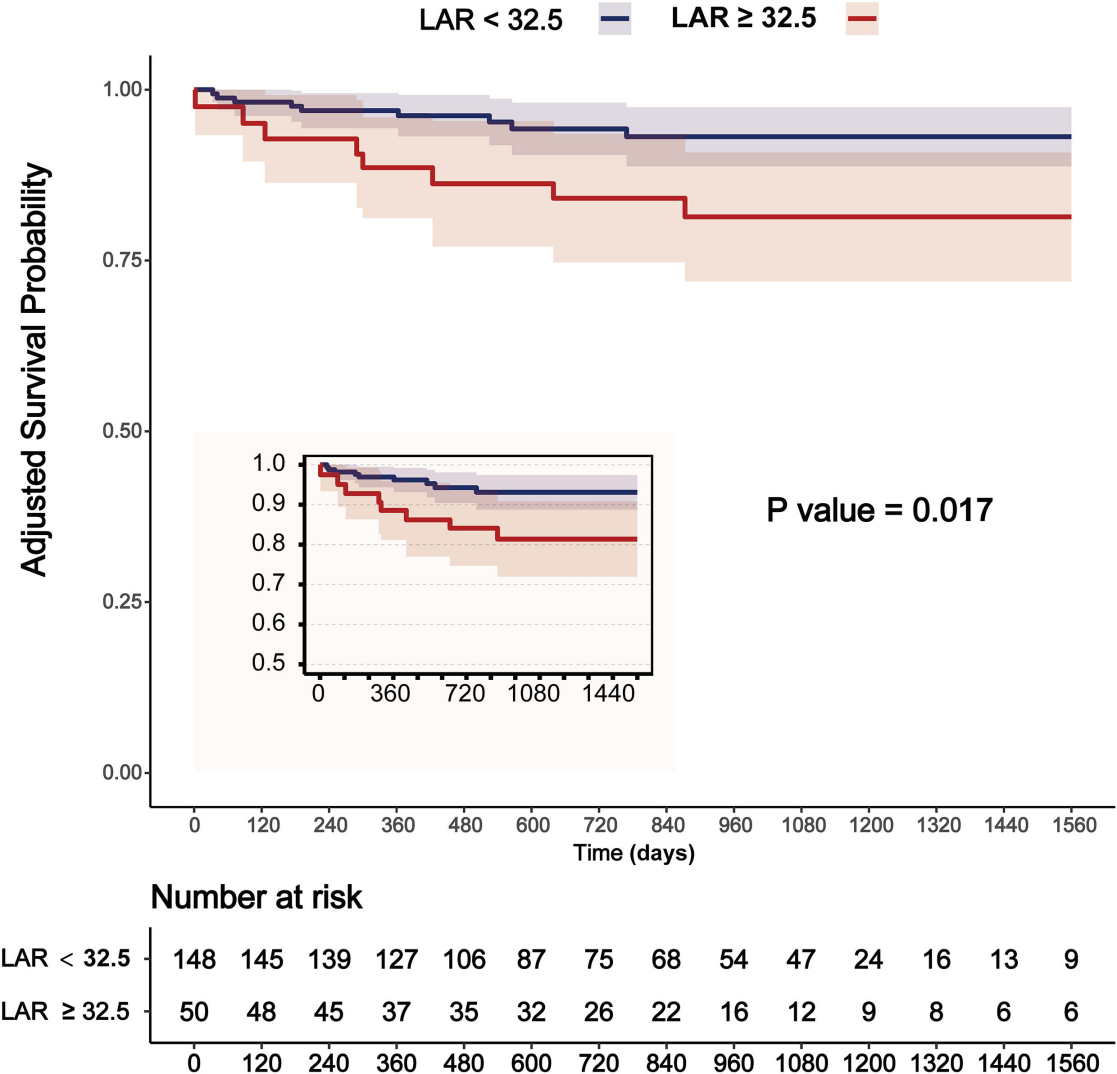
Adjusted Kaplan-Meier curves for ACM with the LAR cutoff of 32.5 mg/g. Adjusted for age + sex + BMI + DM + DLP + PAD + CRP + Statins. Unit of LAR: mg/g.

**Figure 3** presents the adjusted K-M survival curves comparing patients with a low LAR (< 32.5 mg/g) to those with a high LAR (≥ 32.5 mg/g) over a 4-year follow-up period. Notably, A high LAR (≥ 32.5 mg/g) was significantly associated with an increased risk of long-term mortality with adjustment for key clinical covariates (age, sex, BMI, DM, DLP, PAD, CRP, Statins) (*P* = 0.017).

## Discussion

The primary finding of this study is that a high-level LAR is associated with the adjusted risk of ACM in patients with stable CAD after PCI. Specifically, each 1 mg/g increase in LAR is associated with an approximately 6% elevation in the adjusted ACM risk. In the IPTW analyses, after balancing the groups for the fullest set of covariates (age, sex, BMI, DM, DLP, PAD, CRP, and statins), patients with a high LAR (≥ 32.5 mg/g) had a 3.16-fold higher risk of ACM compared to those with a low LAR (95% CI 1.25–7.97, *P* = 0.028). Over a 4-year follow-up period, individuals with a high LAR (≥ 32.5 mg/g) have a lower adjusted survival probability than those with a relatively low LAR (< 32.5 mg/g) for ACM outcome. In a separate analysis, LAR ≥ 32.5 mg/g is driven by elevated levels of atherogenic lipids (LDL-C, TC) and reduced serum albumin, whereas male sex exerts a protective effect.

A high baseline LDL-C level was not associated with a reduced risk of clinical outcomes in patients with CAD undergoing their first PCI (19). Notably, higher LDL-C levels are linked to increased rates of cardiovascular events (20). A large-sample, real-world study demonstrated that LDL-C and high-sensitivity CRP jointly mediated the effect of lipoprotein(a) [Lp(a)] on outcomes in CAD patients after PCI; specifically, when LDL-C was well controlled, the adverse effect of elevated Lp(a) on cardiovascular risk appeared to diminish regardless of hs-CRP levels (21). Another large cohort observational study found that the risk of major adverse cardiac and cerebrovascular events in post-PCI CAD patients with an LDL-C/HDL-C ratio > 2.93 was 1.455 times higher than that in those with a ratio < 2.12 (22). Restricted cubic spline analysis from a separate large cohort observational study revealed a U-shaped relationship between the non-HDL-C/HDL-C ratio (NHHR) and major adverse cardiac and cerebrovascular events (23). In contrast, total TC/HDL-C cannot predict outcomes (24). As shown in a large-scale population-based observational study (25), among 47,884 included patients who underwent PCI, after a median follow-up of 3.2 years, compared with the LDL-C < 70 mg/dL group, the adjusted HRs for cardiovascular events were 1.17 (95% CI: 1.09–1.26) in the 70 to < 100 mg/dL group and 1.78 (95% CI: 1.64–1.94) in the ≥ 100 mg/dL group. Collectively, these findings indicate that high LDL-C levels constitute a crucial prerequisite for these composite indicators or combinations of indicators to exert an influence on prognosis after PCI. Our study demonstrated that the LAR, a novel composite index including LDL-C, is associated with ACM risk in patients with stable CAD after PCI during a 4-year follow-up.

Serum albumin level has long been recognized as a significant predictor of adverse events following PCI across various etiologies (8, 9, 26–29), Additionally, glycosylated serum albumin (30) and ischemia-modified albumin (31) are also associated with adverse events after PCI, and changes in serum albumin levels over time predict outcomes following PCI (32), Therefore, we chose to investigate the LAR, a composite index incorporating albumin, and found that a high-level LAR (≥ 32.5 mg/g) was associated with ACM in PCI patients during a 4-year follow-up.

Although numerous albumin-related indices—such as the uric acid/albumin ratio (33), platelet/albumin ratio (34), CRP/albumin (35), and fibrinogen/albumin ratio (36)—have demonstrated prognostic significance in PCI patients with various conditions, it is evident that LDL-C remains a key target for prioritized management in CAD patients undergoing PCI (37). In this study, LAR, as a novel index, holds important clinical implications. Elevated LAR—whether analyzed as a continuous variable or using a cutoff value of 32.5 mg/g—was confirmed to be associated with ACM.

Despite some evidence that utilization of drug-eluting stent (DES) has predominantly benefited women (38, 39), most studies have yielded conflicting results on long-term sex-related outcomes following PCI (40–42), while in an early large-scale, individual patient data pooled analysis of contemporary PCI trials, women had a higher risk of major adverse cardiovascular events and ischemia-driven target lesion revascularization (ID-TLR) compared with men at 5 years following PCI (43). In our study, male patients exhibited a lower LAR, and male sex was identified as an independent protective factor against elevated LAR. Given the heterogeneity in existing findings regarding sex differences, further studies are warranted to validate the association between sex and elevated LAR risk, as well as its impact on PCL prognosis.

This study has several limitations. First, it is a single-center retrospective study with a small sample size, of which only 50 patients were allocated to the high-LAR group. The limited number of clinical endpoint events raises concerns regarding the stability of the multivariable Cox regression models and introduces a potential risk of model overfitting. Data on dietary intake and eating habits were not collected, which may limit the ability to adjust for potential confounding factors. Second, the derivation of the LAR cutoff (32.5 mg/g) by dividing the clinical thresholds of LDL-C and albumin appears largely data-driven and lacks external validation. While a trend test was utilized in the present study, the optimal LAR cutoff value would have been more precisely delineated via ROC curve analysis or Youden Index-based assessment in a larger sample cohort, a limitation that merits further investigation. Third, although this study found that a high level of LAR is associated with ACM in CAD patients following PCI, thereby providing potential additional prognostic insights, it cannot address the question of whether LAR is superior or inferior to individual indicators such as LDL-C or albumin, nor can it definitively clarify whether LAR offers a tangible predictive incremental value over these markers. Fourth, the study population is exclusively composed of Japanese patients from a single center. Given that lipid profiles, albumin levels, and PCI outcomes may vary across ethnicities and healthcare settings, it is important to note that the association between baseline LAR and ACM risk is specific to this patient cohort, limiting the generalizability of the findings to broader populations. Previous study showed that LAR cannot be used to predict proteinuria in patients with HbA1C levels above 10.0% (44).

Nevertheless, to minimize potential confounding bias, the current study employed consistent covariate adjustment across multiple analytical approaches, including IPTW analysis, and adjusted Kaplan-Meier survival curves. What is more, we have adjusted for statin therapy as a confounding therapeutic factor. The standardized adjustment strategy enhances the internal validity and generalizability of the study findings to a certain extent.

## Conclusions

High level LAR is associated with ACM in stable CAD patients following PCI, with a level above 32.5 mg/g distinguishing a high-risk subgroup that warrants closer clinical monitoring due to a significantly elevated risk of adverse outcomes. Driving LDL-C to the target level is a cornerstone of management. For patients with low albumin: A prompt evaluation to identify the cause (e.g., inflammation, malnutrition, chronic disease) should be initiated, followed by targeted interventions such as nutritional support and optimization of underlying conditions to mitigate LAR-related risk.

## Data Availability

Publicly available datasets were analyzed in this study. This data can be found here: https://doi.org/10.5061/dryad.fn6730j.
